# Explantation of Pedicle Screws: When, Why, and How?

**DOI:** 10.7759/cureus.71711

**Published:** 2024-10-17

**Authors:** Vladimir S Prandzhev, Donika I Vezirska

**Affiliations:** 1 Department of Neurosurgery, Military Medical Academy, Sofia, BGR

**Keywords:** pedicle screw fixation, pedicle screw removal technique, screw extraction, screw failure, screw removal

## Abstract

The insertion of pedicle screws is one of the most common procedures in neurosurgical spinal interventions. It has been used for the fixation and immobilization of spinal segments secondary to trauma or as part of complex spinal reconstruction for vertebral metastatic disease, degenerative disease, or infection. However, increasing rates of pedicle screw use may also be the cause of more frequent revision surgery. The stabilization system may become loosened or defective, which could cause instability of the segment, severe neurological deficit, and intense pain. In addition to this, the screws may be removed in case of satisfactory consolidation depending on the individual decision of the surgeon. Despite the widespread use of pedicle screw fixation, there is limited research regarding the precise circumstances that may cause the need to remove the stabilization system. To our knowledge, this is the first research item to review the causes and the exact time for removal of inserted pedicle screws, as well as to outline techniques for the explantation and the possible short- and long-term outcomes after the procedure.

## Introduction and background

Currently, pedicle screw insertion is one of the most common neurosurgical interventions. The segment that is most often operated on is the thoracolumbar junction (T10-L2) [1,2]. This is believed to be because of the anatomical considerations of this region as a transition area - the fixed and less mobile thoracic spine with the attached ribs to the more flexible lumbar spine. Another frequently affected segment is the lumbosacral region. Pedicle screw fixation is frequently used in association with laminectomy for decompression in spinal trauma with spondylolisthesis or burst fractures of the vertebrae [2-4]. Screws may or may not be implanted along with an interbody cage to achieve fusion of the segment [5].

However, the need for revision surgery may occur for numerous reasons, i.e. adjacent segment disease, pseudarthrosis, and implant failure [6,7]. This often necessitates the explantation of the stabilization system and, more specifically, the pedicle screws that are pivotal to the fixation [7]. Additionally, some surgeons prefer to remove the fixation when the healing process of the fracture is complete and there is sufficient consolidation of the segment [8].

In this article, we shortly review the possible causes for the explantation of pedicle screws, as well as the precise time to perform the removal. Additionally, we assess different techniques for pedicle screw explantation that may aid the surgeon in this occasionally challenging task and discuss possible short- and long-term outcomes after screw removal.

## Review

Methodology of the review

A narrative review was conducted on the online database PubMed. We used the keywords “pedicle screw removal”, “pedicle screw explantation”, “fixation implant removal”, and “pedicle screw removal technique”. Search results were not limited to a specific time period; however, most of the works we found were published after 2015. Our search criteria are shown in Table 1.

**Table 1:** Inclusion and exclusion criteria for the relevant articles

Inclusion criteria	Exclusion criteria
Specific focus of the article on causes, time, and/or techniques for pedicle screw explantation	Focus other than pedicle screw explantation
Articles written exclusively in the English language	Papers not available in full-text and/or available as abstract only
Human studies only	Animal studies

Results

**Figure 1:**
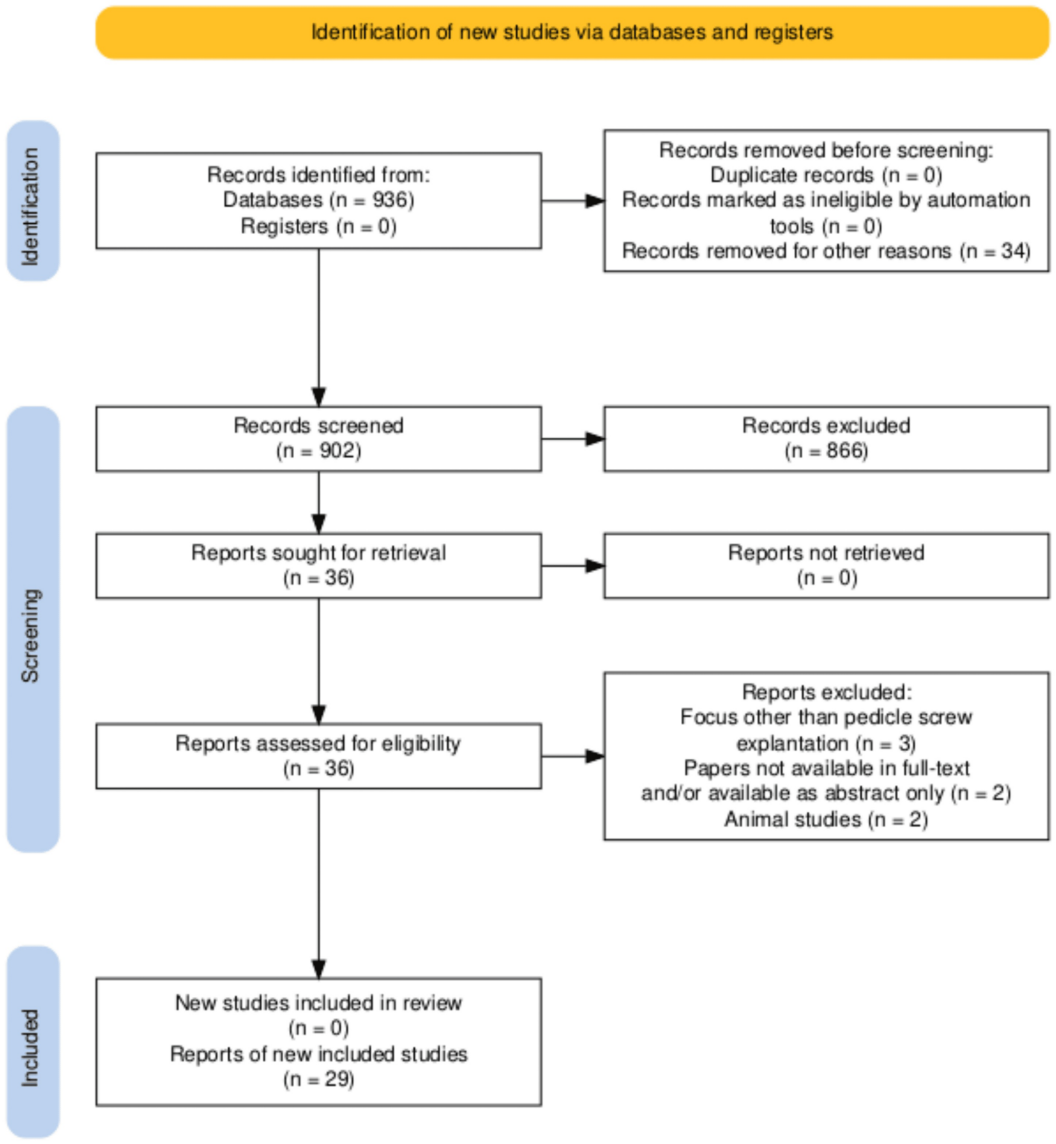
PRISMA flowchart of the search process PRISMA: Preferred Reporting Items for Systematic Reviews and Meta-Analyses

Our search resulted in 29 articles that fulfilled the aforementioned criteria; the search process is illustrated by the Preferred Reporting Items for Systematic Reviews and Meta-Analyses (PRISMA) flowchart in Figure 1.

We created a table with the data derived from the search, grouping the results by three main topics - causes for screw removal, screw removal techniques, and effects after pedicle screw removal. Our search gathered 20 articles that include causes that may necessitate fixation system removal and outcomes that follow this procedure. From the data therein, we created Table 2.

**Table 2:** Possible causes for and outcomes after pedicle screw fixation Sources for causes: [6-17] Sources for outcomes: [18-25]

Groups	Occurrences
Causes	Surgical site infections (SSIs), local pain, protrusion of the implanted system, hypersensitivity reactions, growth disturbance, screw misplacement, screw breakage, screw loosening
Outcomes	Effect on range of segmental motion, alleviation of pain, secondary kyphosis, compression fracture, recurrent instability, lumbar disc herniation, implant removal-related complications

Our search found nine articles (seven technical notes, one original article, and one case report) that describe different screw removal techniques in different case scenarios (Table 3).

**Table 3:** Brief description of the pedicle screw removal techniques Sources: [26-34]

Authors	Article type	Brief description of the method
Donovan et al. (1996) [26]	Technical note	Extracting a transdural screw that avulsed L1 nerve root and pierced through the dura
Duncan et al. (1998) [27]	Technical note	Drilling a pilot hole with a side-cutting bit attached to a high-speed drill next to the long axis of the fragment in the superior aspect of the pedicle and engaging the threads of the screw
Di Lorenzo et al. (2000) [28]	Technical note	Engaging the head of the broken screw with a drill bit attached to a slow-speed drill
Ulus et al. (2010) [29]	Technical note	Placing a small piece of a rod at the head of the screw and attaching it firmly with a nut to tighten the head of the polyaxial screw, then removing it like a mono-axial screw using a big clamp or needle driver
Elmadağ et al. (2015) [30]	Technical note	Removing the head of the screw, putting the screw in the U-section of the described tool, tightening the cap screw, and fixing the screw in the end section of the apparatus; the polyaxial screw becomes monoaxial and can be removed by turning the handles on the tool’s shaft
Zhang et al. (2019) [31]	Original article	Grinding both sides of the nut between the two arms of the nail tail with a dental drill, forming an “I” type notch, loosening the nut with a suitable “I” type screwdriver or a bone blade; fixing 1-cm cut titanium rod in the groove of the end of the screw, then consolidating the nail tail and the nail body into one, clamping the two arms of the screw by locking pliers and turning them to remove the screw
Shahzad et al. (2021) [32]	Technical note	Curetting the newly formed bone, using a 2mm burr to expose 30% of the circumference of the superior aspect of the screw (at least two threads exposed to accommodate a double loop of Vicryl-1 around them), pushing each subsequent screw thread with a Swedish dissector 7/8′′ 200mm sequentially out of the pedicle
Zhang et al. (2023) [33]	Original article	Reusing the rod, cutting it off to fit the groove of the counter-torque (with a length slightly longer than the groove), and turning the polyaxial screw into a monoaxial one
Singh et al. (2023) [34]	Case report	Using a jumbo cutter to cut the rods close to the head of the screw, turning the polyaxial screw into a monoaxial one

Discussion

Causes for Screw Removal

The causes for screw removal may be urgent or elective. Often, the sole reason for the explantation is local discomfort and pain, which the patient associates with the fixation system [7]. This is also the primary reason for pedicle screw explantation. However, this may not always be the cause of the discomfort and the surgeon should discuss the possibility of an unsatisfactory outcome of the surgery. Careful assessment of the neurological symptoms and imaging diagnostics should be carried out before deciding on the screw removal. For example, obvious radiologic data for pedicle screw misplacement with a neurological deficit is an indication for revision surgery.

Aseptic or septic inflammation surrounding the pedicle screw is a typical reason for screw loosening necessitating explantation. Agarwal et al. describe the appearance of dormant biofilm from the implants in the initial surgery as a possible reason for the occult infection phenomenon, which presents as a local process, the bone disassociates from the implant and the screws loosen, resulting in aseptic pseudoarthrosis [9]. Forms of prevention of this problem may be the change of gloves immediately before the implantation of the screws, Keller funnels, or wound protectors [6]. A case report by Botolin et al. notes that metal wear particles from the titanium implants generated by micromotions play a role in screw loosening, which is further proven by the lack of microorganisms from the microbiological probe of their patient. The patient presented with a painful paraspinal soft tissue mass, which was suspicious for infection but no microorganisms from the microbiological probe were identified [10].

However, septic inflammation and surgical site infections (SSIs) are issues that should be managed in a relevantly urgent manner because they may transform into deeper tissue infections and subsequently lead to septic complications [11,12]. Microorganisms from the *Staphylococcus* and *Propionibacterium* genus are the most frequently identified to cause these infections. The intraoperative application of vancomycin powder may be relevant in the context of SSI prevention. Early postoperative infection can be managed conservatively but 4-6 weeks of antibiotic treatment was indicated for optimal results [12]. The use of polymethylmethacrylate (PMMA) as a means to strengthen the construction in osteoporotic patients was studied to compare infection rates between cemented and non-cemented constructs; no significant difference was found [13].

Other factors that influence the rate of screw loosening include bilateral facet joint removal, number of fused levels, and bone density; furthermore, increment in outer diameter, decrease in core diameter, and helical pitch decrease this risk [14]. Growth disturbance in growing young individuals may also necessitate screw removal in order not to stunt the development [7]. Cox et al. demonstrate the feasibility of temporary internal fixation in young adults with subsequent removal of the fixation system to preserve segment mobility [15].

Bony chance fractures are most commonly managed conservatively (with bedrest and orthosis or a cast) but severe pain that cannot be managed via medication may be an indication for temporary internal fixation without arthrodesis [15,16]. According to Cox et al., six months is sufficient for the proper healing of the fracture in young adults. After plain radiograph verification of the fixed vertebrae, the pedicle screws may be removed [15]. A large case series by Ding et al. recommends plain radiograph control of the fractured vertebrae consolidation every three months to decide on the stabilization system explantation [16].

Radiological imaging is crucial to the decision to remove the pedicle screws. Radiolucency surrounding the screw on MRI and CT imaging is indicative of screw loosening [10,17]. Kanayama et al. note that the presence of MRI findings of vertebral osteomyelitis and/or intervertebral abscess necessitate screw removal. If this timestamp for explantation is missed and only conservative treatment is applied, loss of stability and vertebral bone compromise may progress, thus rendering revision surgery obsolete at later stages [12].

Removal Techniques [26-34]

A common challenge for spine surgeons is a fixation system for which they do not have the instrumentation set. The removal of implants, precisely screws that are known to be well-fixed into the pedicles with the aid of newly formed bony tissue, is an impossible task without the appropriate instruments. The majority of articles on the matter propose the use of a cut rod to turn the polyaxial screw into a monoaxial one and turn either the two ends of the rod (using them as two ends of a lever) or the head of the screw with pliers or a strong needle holder [29,33]. This preserves the pedicle and allows for the insertion of another screw with a slightly different diameter if need be.

This is a method that is rendered obsolete if the head of the screw is broken. In this case, Duncan et al. suggest drilling with a side-cutting bit attached to a high-speed drill next to the pedicle screw, which would facilitate engaging its threads [27]. However, this may compromise the pedicle and decreases the chances for a strong purchase of another screw on the same side and level if one is needed.

What Happens After Implant Removal? [7,18]

A systematic review by Visagan et al. outlines the most visible effect of screw removal - a significant decrease in back pain [8]. However, the study is based on minimally invasive percutaneous screw insertion, which does not require midline incision and soft tissue dissection and retraction. Revision open surgery is linked to higher perioperative risks in comparison to minimally invasive alternatives such as wound healing issues, infection, and soft tissue ischemia with subsequent necrosis.

The range of segmental motion can be regained if the implants are removed in a maximum of two years after the initial surgery. On average, most studies recommend that explantation be carried out at least one year after the surgery [8,16,18,19]. However, after this period, the range decreases significantly and fixation system removal becomes irrelevant if no clinical symptoms arise.

Segment stiffness may lead to adjacent segment disease and cause mechanical stress [20]. Still, the removal of screws increases the axial load on the fused segments, which could transfer onto the adjacent segments and result in disc herniations at a lower level and compression fractures [21,22]. Some studies show that screw removal may cause adjacent segment disease of the upper levels in the osteoporotic spine [23]. Liu et al. report the presence of recurrent instability in certain cases with incomplete fusion or pseudoarthrosis [24].

Secondary kyphosis is another possible outcome after screw removal. According to Hirahata et al., one could predict this deformity via the vacuum phenomenon of the intervertebral disc, which may be present in CT imaging, thus evaluating the possibility of fixation system removal [25]. Another study by Oh et al. confirms the role of intervertebral disc collapse present on MRI imaging in the formation of secondary kyphosis and vertebral collapse [26].

Limitations

A major limitation of this review is the very limited number of articles with large case series to include; this may lower its level of evidence. Another hindrance is the lack of long-term observations of the patients who underwent hardware removal and the ones who did not. This matter may have to be researched more thoroughly to achieve a clearer perspective of the advantages and disadvantages of this procedure.

## Conclusions

Pedicle screw fixation with or without the implantation of an interbody cage is a neurosurgical intervention that allows for the consolidation of fractures and the prevention of deformity. However, oftentimes, it is necessary to remove the implanted screws because of different reasons, such as implant failure and infection, or simply because of significant patient discomfort and local pain. It could also be an elective procedure resulting from the surgeon’s decision to explant the screws after a satisfactory healing of the fracture; this could prevent adjacent segment disease and it could regain segmental motion range if carried out promptly.

Currently, there have been fervent discussions as to the necessity to explant pedicle screws. It has been generally agreed upon that the optimal time for elective removal is one year after the fixation, although radiographic verification of the segment is crucial to the final decision. The time for symptomatic pedicle screw issues, such as neurological deficit, severe pain, and infection, is as soon as possible. Nevertheless, further research and larger case studies are to be carried out to refine surgical decision-making.
